# Rationale and protocol design of a phase II study of first-line osimertinib treatment for patients with poor performance status and EGFR mutation-positive non-small cell lung cancer (OPEN/TORG2040)

**DOI:** 10.1186/s12885-022-10409-6

**Published:** 2022-12-15

**Authors:** Tomoya Fukui, Jiichiro Sasaki, Satoshi Igawa, Akiko Kada, Toshiki I. Saito, Yoshihito Kogure, Hiroaki Okamoto, Katsuhiko Naoki

**Affiliations:** 1 Department of Respiratory Medicine, Kitasato University School of Medicine, 1-15-1 Kitasato, Minami-ku, Sagamihara, Kanagawa 252-0374 Japan; 2 Research and Development Center for New Medical Frontiers, Kitasato University School of Medicine, Kanagawa, Japan; 3 Clinical Research Center, National Hospital Organization Nagoya Medical Center, Aichi, Japan; 4 Department of Respiratory Medicine, National Hospital Organization Nagoya Medical Center, Aichi, Japan; 5 Department of Respiratory Medicine and Medical Oncology, Yokohama Municipal Citizen’s Hospital, Kanagawa, Japan

**Keywords:** Osimertinib, Epidermal growth factor receptor, Non-small cell lung cancer, Poor performance status, First-line chemotherapy

## Abstract

**Background:**

Cancer chemotherapy indications for patients with poor performance status and advanced lung cancer are limited. Molecular targeted drugs, including epidermal growth factor receptor (EGFR)-tyrosine kinase inhibitors, can be used in patients with poor performance status owing to their high efficacy and safety. The third-generation EGFR-tyrosine kinase inhibitor osimertinib has demonstrated effectiveness in the initial treatment of advanced EGFR mutation-positive non-small cell lung cancer in patients with good performance status; however, no evidence exists of the drug’s effectiveness in patients with poor performance status in a prospective study. We designed a study that aims to investigate the efficacy and safety of first-line osimertinib treatment in patients with advanced non-small cell lung cancer harboring sensitive EGFR mutations and with poor performance status.

**Methods:**

The OPEN/TORG2040 study is a multicenter, single-arm, phase II trial for patients with unresectable, advanced EGFR mutation-positive non-small cell lung cancer with a poor performance status (≥ 2). Eligible patients will receive osimertinib until disease progression or unacceptable toxicity. The primary endpoint is the objective response rate of the first-line osimertinib treatment. Considering a threshold value of 45%, expected value of 70% for objective response rate, one-sided significance level of 5%, statistical power of 80%, and ineligible patients, the sample size was set to 30. The secondary endpoints are disease control rate, performance status improvement rate, and safety and patient-reported outcomes using the European Organization for Research and Treatment of Cancer Quality of Life Questionnaire-Core Quality of Life Questionnaire and Lung Cancer 13. Time to treatment failure, progression-free survival, and overall survival will also be assessed.

**Discussion:**

Our study can determine the clinical benefits of osimertinib treatment in patients with poor performance status, since the clinical outcomes of patients with EGFR mutation-positive non-small cell lung cancer with poor performance status treated with this drug as a first-line treatment have not been sufficiently evaluated.

**Trial registration:**

Japan Registry of Clinical Trials: jRCTs041200100 (registration date: February 12, 2021).

## Background

Epidermal growth factor receptor (EGFR) gene mutations have been identified as predictors of the effect of EGFR-tyrosine kinase inhibitors (TKIs) on EGFR mutation-positive non-small cell lung cancer (NSCLC) [1–3]. Among the EGFR gene mutations, exon 19 deletions (DEL) and exon 21 L858R point mutations, which account for approximately 90% of the total mutations, and uncommon mutations, such as exon 18 G719X and exon 21 L861Q, are known to be EGFR-TKI-sensitizing mutations [4, 5]. In addition, a T790M mutation has been identified in approximately half of the patients as a resistance factor to first- and second-generation EGFR-TKIs, such as gefitinib, erlotinib, afatinib and dacomitinib.

Osimertinib is a third-generation, irreversible EGFR-TKI that selectively inhibits both EGFR-TKI-sensitizing and EGFR-T790M-resistant mutations [6, 7]. In a double-blind, phase III trial (FLAURA), osimertinib showed efficacy superior to that of first-generation gefitinib or erlotinib in the first-line treatment of EGFR mutation (DEL or L858R)-positive advanced NSCLC, with a similar safety profile and lower rates of serious adverse events [8, 9]. Osimertinib is currently being used as the first-line treatment for patients with advanced EGFR mutation-positive NSCLC in clinical practice. However, in the FLAURA trial, patients with a performance status (PS) of 0 to 1 were eligible, and the efficacy and safety of osimertinib treatment in patients with EGFR mutation-positive NSCLC with a poor PS (2 or more) have not been fully verified.

Poor PS is a known adverse prognostic factor in advanced NSCLC [10, 11] and a risk factor for serious adverse events, including drug-induced interstitial lung disease [12]. The median survival of patients with advanced NSCLC who are not eligible for cytotoxic chemotherapy due to poor PS was reported about 3–4 months [13, 14]. A previous prospective study of first-line gefitinib treatment for patients with advanced EGFR mutation (DEL, L858R, G719X or L861Q)-positive NSCLC without indication for cytotoxic chemotherapy as a result of poor PS has supported the use of EGFR-TKIs for these patients [15]. Several studies have indicated that osimertinib could be beneficial in patients with poor PS and EGFR T790M mutation-positive NSCLC following the progression of first- and second-generation EGFR-TKI treatments [16–19]. However, the existing data are insufficient to determine the efficacy and safety of osimertinib in chemo-naïve patients with EGFR mutation-positive NSCLC and poor PS.

First-line osimertinib treatment for patients with poor PS could be beneficial and clinically meaningful, considering the limited benefits of cytotoxic chemotherapy. Thus, this study aims to assess the efficacy and safety of first-line osimertinib treatment in patients with poor PS and advanced NSCLC harboring sensitizing EGFR mutations.

## Design

### Study design and treatment

**Fig. 1:**
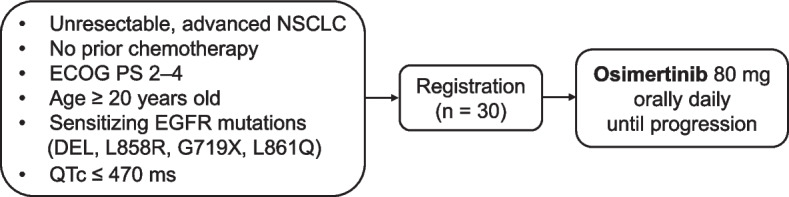
OPEN/TORG2040 study design. *NSCLC* non-small cell lung cancer, *ECOG* Eastern Cooperative Oncology Group, *PS* performance status, *EGFR* epidermal growth factor receptor, *DEL* deletion, *QTc* corrected QT

This study, whose objective is to assess the efficacy and safety of first-line osimertinib treatment in patients with advanced NSCLC harboring sensitive EGFR mutations and with poor PS, was designed as a multicenter, single-arm, phase II trial conducted by the Thoracic Oncology Research Group (TORG) in accordance with the Declaration of Helsinki. The study schema is illustrated in Fig. 1. The protocol was approved by the National Hospital Organization Nagoya Medical Center Certified Review Board of Clinical Research (approval date: December 21, 2020; approval number: C2020-010). This clinical trial was registered in the Japan Registry of Clinical Trials (registration date: February 12, 2021; registry number: jRCTs041200100). Registration for this study begin in February 2021 and will end in August 2022. Eligible patients with EGFR mutation-positive NSCLC and with a PS of 2 to 4 will receive osimertinib (80 mg orally, once daily) until disease progression or unacceptable toxicity. However, if the patients continue to show clinical benefit with the treatment as judged by the investigator, they may continue to receive osimertinib beyond the progression defined by the Response Evaluation Criteria in Solid Tumors (RECIST) version 1.1.

### Eligibility criteria

Each patient’s general condition will be assessed using the Eastern Cooperative Oncology Group PS, and patients with PS ≥ 2 will be eligible for this study. The key patient inclusion and exclusion criteria are listed in Table 1. After their eligibility is confirmed, the patients will be asked to provide informed consent. Patient registration began in February 2021 and should continue for 1.5 years.

**Table 1:** Key eligibility criteria

Inclusion criteria	
1	Histologically or cytologically proven NSCLC
2	NSCLC with sensitizing EGFR mutations (exon 18 G719X, exon 19 deletions, exon 21 L858R, and exon 21 L861Q)
3	Unresectable NSCLC with stage III/IV cancer or postoperative recurrence
4	Age 20 years or older
5	ECOG PS 2–4 owing to NSCLC and life expectancy of more than 12 weeks
6	At least one measurable lesion according to RECIST version 1.1
7	No prior cytotoxic chemotherapy, immune-checkpoint inhibitor, or targeted agent, including EGFR-tyrosine kinase inhibitor
8	Adequate laboratory data within 14 days before registration
	a) Neutrophil count ≥1500/mm^3^
	b) Hemoglobin ≥9.0 g/dL
	c) Platelet count ≥100,000/mm^3^
	d) Aspartate aminotransferase ≤2.5 times the normal value upper limit
	e) Alanine aminotransferase ≤2.5 times the normal value upper limit
	f) Total bilirubin ≤1.5 times the normal value upper limit
	g) Creatinine ≤1.5 times the normal value upper limit or creatinine clearance < 50 mL/min (Cockcroft–Gault formula)
	h) SpO_2_ ≥ 90% (with or without oxygen therapy)
9	Written informed consent
**Exclusion criteria**	
1	EGFR exon 20 insertions
2	Synchronous or metachronous active double malignancies
3	History of interstitial lung disease, drug-induced pneumonitis, radiation pneumonitis requiring systemic steroid therapy, or active interstitial pneumonitis
4	Ingestion not possible or refractory nausea and vomiting
5	Presence of active infections requiring antibiotics
6	Severe psychological disorder
7	Active brain metastasis
8	Serious uncontrolled medical conditions
9	Systemic administration of steroids for 4 weeks or longer (prednisolone-equivalent doses within 20 mg/day are allowed)
10	ECOG PS reduction due to comorbidities
11	Major surgery or radiation therapy with more than 30% of bone marrow or widespread radiation within 4 weeks prior to enrollment
12	QT prolongation on resting electrocardiogram (QTc ≥ 470 ms)
13	History of hypersensitivity to osimertinib
14	Pregnant, currently breastfeeding, positive pregnancy test results, or unwilling to practice contraception during this study
15	Other conditions not suitable for this study

*NSCLC* non-small cell lung cancer, *EGFR* epidermal growth factor receptor, *ECOG* Eastern Cooperative Oncology Group, *PS* performance status, *RECIST* Response Evaluation Criteria in Solid Tumors, *QTc* corrected QT

### Evaluation of treatment efficacy

Computed tomography scans of the chest and abdomen, a computed tomography or magnetic resonance imaging scan of the brain, a bone scan or positron emission tomography scan, blood test, urine test, electrocardiography, and echocardiography are required before initiation of study treatment. Patients will undergo a tumor assessment, Eastern Cooperative Oncology Group PS evaluation, and patient-reported outcome assessments using the European Organization for Research and Treatment of Cancer (EORTC) Quality of Life Questionnaire (QLQ)-Core Quality of Life Questionnaire (C30) and Lung Cancer 13 (LC13) at baseline, every 6 weeks (± 2) during the first 24 weeks, and every 8 weeks (± 2) thereafter. The tumor response will be evaluated in accordance with RECIST version 1.1. Adverse events will be recorded using the National Cancer Institute’s Common Terminology Criteria for Adverse Events version 5.0.

### Endpoints

The primary endpoint is the objective response rate (ORR) of first-line osimertinib treatment. ORR is defined as the proportion of patients whose best response is complete response or partial response. However, response evaluation following the initiation of other anticancer treatments is not employed.

The secondary endpoints are disease control rate, proportion of PS improvement, safety, and patient-reported outcomes. In addition, the time to treatment failure, progression-free survival, and overall survival will also be assessed. Disease control rate is the proportion of patients whose best response is complete response, partial response, or stable disease. Proportion of PS improvement is defined as the proportion of patients who have improved by one or more steps from baseline after the start of study treatment. The time to treatment failure is defined as the duration from registration to the first date of disease progression, death, or stop of the trial treatment. Progression without diagnostic imaging is also included in disease progression. Survivors who have not progressed and continue treatment on their last visit will be censored at the last date of observation. Progression-free survival is defined as duration from registration to the confirmation of disease progression or the date of death. A survivor without disease progression is censored at the date of evaluation based on RECIST. Overall survival is the duration from registration to all causes of death. A survivor is censored at the last date of observation.

### Statistical analysis

A previous Japanese study for patients with EGFR mutation-positive NSCLC without indication for cytotoxic chemotherapy as a result of poor PS, which included 26 of 30 patients with PS 2 to 4, reported that first-line gefitinib had an ORR of 66% (90% confidence interval [CI], 51 to 80%) [15]. Retrospective studies of first-line or second-line EGFR-TKI treatment for patients with EGFR mutation-positive NSCLC with PS 2 to 4 showed an ORR of 16.4 to 70.0% [13, 16, 17, 20]. Although there was insufficient evidence for first-line osimertinib in EGFR mutation-positive patients with poor PS, our prospective observational study reported an ORR of 56.3% (95% CI: 47.1 to 78.0%) [21]. This study is to confirm similar efficacy of gefitinib monotherapy and safety; therefore, the threshold of ORR was set to 45%. In the Japanese PS 0 to 1 population of the FLAURA trial, the ORRs of osimertinib and gefitinib were 75.4 and 76.4%, respectively [9]. Since the patients in this study should have PS 2 to 4, the expected ORR was set to 70%. With a threshold value of 45% and expected value of 70% for ORR, one-sided significance level of 5%, and statistical power of 80%, and by taking ineligible patients into account, the sample size was set to 30 patients.

The patients who meet the key eligible criteria and receive at least one trial treatment will comprise the full-analysis set (FAS). Within the FAS, an analysis set that does not have a serious protocol violation is defined as the per-protocol set. FAS will be used for the primary efficacy analysis. All patients who receive at least one trial treatment will be analyzed for safety.

The exact 90% CI of ORR will be estimated using the Clopper–Pearson method. A binomial test with a one-sided significance level of 0.05 will be performed based on the null hypothesis that the ORR is below the 45% threshold. The 95% CI for disease control rate and proportion of PS improvement will be estimated. The percent of change from baseline for EORTC QLQ-C30 and LC13 will be calculated. The worst grade of adverse event will be summarized. The survival curves for time to treatment failure, progression-free survival, and overall survival will be estimated using the Kaplan–Meier method.

## Discussion

This is the first prospective trial to assess the clinical benefits of osimertinib as a first-line treatment in EGFR mutation-positive NSCLC patients with a poor PS (≥ 2). Patients with poor PS have been excluded from most clinical trials because of their poor prognosis and concerns about management. There is little evidence for first-line osimertinib in EGFR mutation-positive patients with poor PS. Therefore, regarding the setting of the statistical analysis in this study, the threshold of ORR was estimated by referring to the lower limit of the CI for the ORR in the previous retrospective study for patients with EGFR T790M mutation-positive NSCLC and a poor P S[16] and our prospective observational study of first-line osimertinib for patients with poor PS[21]. Molecular targeted therapies, such as EGFR-TKIs, can be expected to promote improvement from the lung cancer-induced deterioration of the physical condition of patients due to their high response rate. Whereas an accumulation of real-world data is important, it is reasonable that a prospective investigation of the efficacy and safety of the treatment, even in patients with poor PS, can be considered useful for clinical practice. The clinical influence of this study will be significant in identifying more effective treatments for bedridden patients with NSCLC to return to their daily lives. The results of the OPEN/TORG2040 study will unveil the clinical benefits of first-line osimertinib treatment in sensitizing patients with EGFR mutation-positive NSCLC and poor PS.

## Data Availability

All data generated or analyzed during this study are included in this published article.

## Abbreviations

EGFR: Epidermal growth factor receptor
TKI: Tyrosine kinase inhibitor
NSCLC: Non-small cell lung cancer
PS: Performance status
RECIST: Response Evaluation Criteria in Solid Tumors
EORTC: European Organization for Research and Treatment of Cancer
QLQ: Quality of Life Questionnaire
C30: Core Quality of Life Questionnaire
LC13: Lung Cancer 13
ORR: Objective response rate
FAS: Full-analysis set
